# Evaluation of Salt Influence on Sugar Consumption by Suspension Cells Based on Spectroscopic Analysis

**DOI:** 10.1155/2013/401718

**Published:** 2013-12-30

**Authors:** Ken-ichiro Suehara, Takaharu Kameoka, Atsushi Hashimoto

**Affiliations:** Division of Sustainable Resource Sciences, Graduate School of Bioresources, Mie University, 1577 Kurimamachiya-cho, Tsu, Mie 514-8507, Japan

## Abstract

The influence of metal salt on sugar consumption by suspension cells in food models constructed by a sugar and salt aqueous solution was investigated based on mid-infrared spectroscopic analysis. The contaminated suspension cells in the food model could be detected using the spectral feature change that measured the present spectrum subtracted in the initial spectrum. The cells were prepared for growth and although the cell did not grow under the induction period, the cell activation (start of sugar metabolism) was detected on the subtracted spectral behavior before the cell growth. The rough grasp of the spectral change behavior is useful for the high-throughput spectroscopic method to detect the contaminated cell activation. Furthermore, the detailed sugar consumption kinetics of the cells was also investigated based on the spectroscopic method. The kind of added salt in the food model influenced the cell activation and the potassium ions play an important role in the plant cells. The living cells activity in fresh food may act to prevent microbial contamination and to suppress the growth of the contaminated microorganism. Both the simple and detailed analyses based on the spectroscopic method presented in this study might be useful for risk management of food.

## 1. Introduction

Microbial contamination is one of the serious problems for risk management of food products. In general, sugar-rich, salt-rich, or fermented foods are processed to prevent microbial contamination. However, consumers tend to like low-salt (sodium-restricted) and low-sugar foods for health care [1, 2]. In this situation, the risk of the microbial contamination will be increased. In addition, the development of sodium-restricted food is required and potassium chloride is used as a food additive for the replacement of sodium chloride. Sodium and potassium balance is one of the important factors for living cell activities because the balance influences the function of the regulation of the osmotic pressure in the cytoplasmic membrane. Therefore, evaluation of the salt influence (environmental change of the surroundings of living cells by salt changing) on the cell activity is important for risk management of food products.

The application of spectroscopy, especially in the infrared region, to these measurements has a high potential. Incidentally, in a parallel study using Fourier transform infrared (FT-IR) spectrometers and attenuated total reflection (ATR) techniques [3, 4], the spectroscopic method using the FT-IR equipped with an ATR accessory (FT-IR/ATR) provided a substantial potential as a quantitative analytical tool for food. Therefore, various FT-IR/ATR spectroscopic methods are under development for the analysis of food and bioproducts [5–13]. We also studied the quantitative analysis of sugar in aqueous solutions using the FT-IR/ATR method [14–17]. In this series of studies, the interaction between saccharide and the aqueous solution with salts using mid-infrared (MIR) spectroscopy was studied because the saccharide-water interaction in the bioproducts is closely related to the other components such as salts [18]. For the five types of alkaline metal chlorides, with a variation of those concentrations, different spectral patterns were found at the absorption bands of OH stretching and bending vibration in the spectra of sugar aqueous solutions. In the fingerprint region spectra, the absorption intensity at the bands of sugar increased with the increase of chloride concentrations. The salt influence on the saccharide spectrum had a similar tendency with potassium chloride and sodium chloride solutions. We could understand the spectral characteristics of the interactions between the sugar and alkaline metal chlorides using the MIR spectroscopy in our previous study [18].

The sugar aqueous solution with salts is one of the simple models used as a food product. However, it is important that the food models are prepared by including the living cells because most of the flesh food will include a living cell in the body, or the exogenous cells will invade the food. We also studied the quantitative analysis and kinetic analysis of sugar metabolism for plant cells in the basal MS salts media [19] using the FT-IR/ATR method [20–27]. The MIR spectrum has shown that the spectral additivity was experimentally applicable for sucrose, glucose, and fructose in the MS media. In addition, we examined the potential of the sugar content determination in the culture media for *Nicotiana tabacum* cv. Bright Yellow Number 2 (TBY-2) cell cultivation using the FT-IR/ATR method by a comparison with the high-performance liquid chromatography (HPLC) method in order to analyze the sugar uptake rate of the TBY-2 cell suspension [21, 22]. We then discovered the importance of understanding the influence of the sugar species on the sugar uptake kinetics during cultivation. The kinetic behavior of the sugar uptake in the TBY-2 cell suspension was dependent on the type of sugar used [26]. In these studies, the salt influence on the sugar uptake by suspension cells is not investigated. However, the cultivation system of this MS salts medium containing sucrose and living TBY-2 cells may be one of the suitable models for food product. In addition, the TBY-2 cells grow under suspension by cell division similar to a microorganism and the cell has the characteristics of the plant cells. Furthermore, the TBY-2 cells produce an ethanol as a fermented product that is similar to many microorganisms [25].

The objective of the present study was to investigate the salt influence on the sugar consumption by suspension cells based on spectroscopic analysis. In the study, we supposed the MS cultivation containing the TBY-2 suspension cells to be a contaminated food model and development of the detection method for the activated cells before the cell growth phase (induction period) was investigated based on the spectral feature change for application of the high-throughput spectroscopy. In addition, more detailed analysis of sugar consumption kinetics of the plant cells when the salt conditions changed was investigated based on the MIR spectral analysis. The activity of the exogenous cells that invade the food will be repressed by the living cells in the food. Evaluation of the salt influence on the sugar consumption of the TBY-2 cells as the plant cells composed in part of the food was investigated and discussed.

## 2. Materials and Methods

### 2.1. Culture Media and Cell Cultivation

The Murashige-Skoog (MS) medium [19], which contained 3% (w/v) sucrose, was used as the food product model and used for suspension cell cultivations. Various types of the modified MS media were prepared to change the salt conditions. The modified MS media, which replaced potassium salt with sodium salt (MS_K→Na), sodium salts with potassium salts (MS_Na→K), all the metal salts with sodium salt (MS_Na), and all the metal salts with potassium salt (MS_K), were prepared (Table 1).

*Nicotiana tabacum* cv. Bright Yellow Number 2 (TBY-2) cells were subcultured in the basal MS salts medium containing 3% (w/v) sucrose and 0.2 mg dm^−3^ 2,4-dichlorophenoxyacetic acid (2,4-D). For the subcultivation, 1.5 cm^3^ of the 7-day-old inoculums in the basal MS medium was inoculated into 95 cm^3^ of a separate fresh medium in 300 cm^3^ flasks, and the precultivation was carried out at 300 K on a rotary shaker (125 rpm) under dark conditions. For the cultivation, cells subcultured for 7 days were washed using the basal MS medium without containing sucrose, and 1.5 cm^3^ of the suspension was inoculated into 95 cm^3^ of separate fresh medium in 300 cm^3^ Erlenmeyer flasks. The cells were grown in the dark under agitation at 125 rpm at 300 K.

### 2.2. Analysis

The dry cell weight in the medium was compared by measuring the turbidity (OD 600 nm). The turbidity was measured by a UV-VIS-NIR scanning spectrophotometer (UV-3100PC; Shimadzu, Kyoto, Japan) using a cuvette with a light path length of 10 mm.

The sugar and ethanol concentrations in the culture medium were determined by the FT-IR/ATR method as described previously [24]. For analysis by MIR spectroscopy, an FT-IR spectrometer (Thermo Fisher Scientific Inc., Nicolet Magna 750, MA, USA) was equipped with a potassium bromide (KBr) beam splitter and a deuterated triglycine sulfate KBr detector was used to obtain the spectra. The ATR spectra were obtained with a horizontal zinc selenide ATR sampling accessory (GRASEBY SPECAC, SPECACLAMP ATR 11080, USA). Sixty-four scans of symmetrical interferograms at 4 cm^−1^ resolution were taken for each spectrum. The ATR spectra of the culture media passed through a 5 *μ*m membrane filter were obtained for 4,000–800 cm^−1^.

### 2.3. Curve Fitting for Cell and Sugar Concentrations

The logistic function expressed by (1) was applied to the plots of the dry cell weight and sugar consumption versus the cultivation time [22, 27]:

(1) $$\begin{array}{c} W=\frac{W_{\text{ini}}-W_{\text{fin}}}{1-e^{\left(\left(t-t_{0}\right)/w\right)}}+W_{\text{fin}}, \end{array}$$

where *W* (g dm^−3^) is the dry cell weight or consumption amount of the sugar. The *t* (d) is the cultivation time. The parameters *t*_0_ and *w*, respectively, denote the inflection point of the time course and the time constant ticking its curve. Moreover, the subscripts ini and fin indicate the value at the initial and final stages, respectively. The growth and sugar consumption rates of the cells were calculated by differentiating (1) with the cultivation time, *t*. In order to discuss the kinetic sugar consumption phenomena while neglecting the cell growth behavior, we attempted to apply the nondimensional cultivation time, *t*_non,*x*_, expressed by [23–25]

(2) $$\begin{array}{c} t_{\text{non},x}=\frac{\left(t-t_{0}\right)}{w_{x}}. \end{array}$$

The nondimensional cultivation time can be calculated using the parameters *t*_0_ and *w*_*x*_, which, respectively, denote the inflection point of the time course of the cell concentration and the time constant ticking its curve, so that the relationship between the specific consumption rate and the nondimensional cultivation time could signify the kinetic sugar consumption characteristics of each sugar cultivation based on the cell growth stage.

## 3. Results and Discussion

### 3.1. Spectral Change during Suspension Cell Cultivation

**Figure 1:**
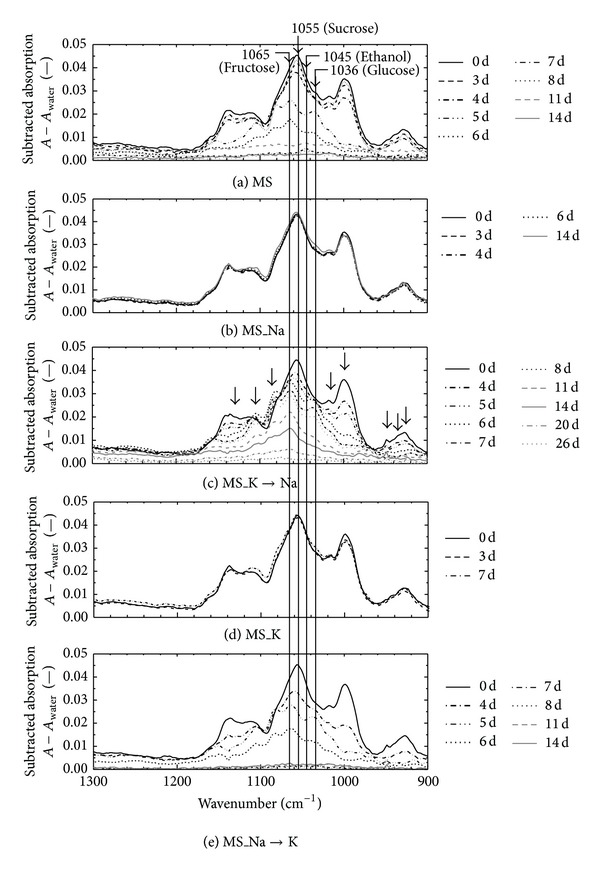
Time behavior of the FT-IR/ATR spectra of the basal MS medium (a) and of the modified MS salt media represented by MS_Na (b), MS_K→Na (c), MS_K (d), and MS_Na→K (e) during cultivations.

Figure 1 shows the FT-IR/ATR spectra during the suspension cell cultivations using the basal MS medium and modified MS media that changed metal salts. In the fingerprint region from 1,300 to 900 cm^−1^, many peaks were observed such as the CO and C–OH stretching modes, which overlapped each other significantly [13, 14, 28], especially in the region from 1,200 to 950 cm^−1^. The peaks depend on the sugar structure and on the interaction between the sugar molecules and their environments. The absorption peaks around 1,055, 1,036, and 1,065 cm^−1^ are characteristic of the sucrose, glucose, and fructose spectra, respectively. The absorption peak around 1045 cm^−1^ is characteristic of the ethanol that was the metabolic product of the suspension cells. These absorptions could be used as the construction of the calibration equation for the simultaneous measurement of the each metabolic material in the media.

During the cultivation, the behavior of the spectral changes was able to be classified in two patterns. The absorbance decreased and the spectral pattern was changed during the cultivation (Figures 1(a), 1(c), and 1(e)). In these cases, the intervals of the spectral feature change were different between the MS_K→Na and MS_Na→K cultivations. The spectral feature change of the MS_Na→K cultivation was similar to that of the basal MS cultivation, but the MS_K→Na cultivation seemed to be different. However, the spectral feature change caused by the sugar composition displayed with an arrow in Figure 1(c) was also observed in the basal MS (Figure 1(a)) and MS_Na→K cultivations (Figure 1(e)). On the other hand, the absorbance was not decreased in the case of the MS_K and MS_Na cultivations although the spectrum pattern was slightly changed (Figures 1(b) and 1(d)). It seems that the environmental change by replacement of the salt significantly influences the sugar metabolism of the suspension cells. In addition, spectral feature changes might be reflected by the activity of the living cells such as the kinetic metabolisms of the cells.

### 3.2. Cell Growth, pH, and Total Sugar Concentration during Suspension Cell Cultivation

**Figure 2:**
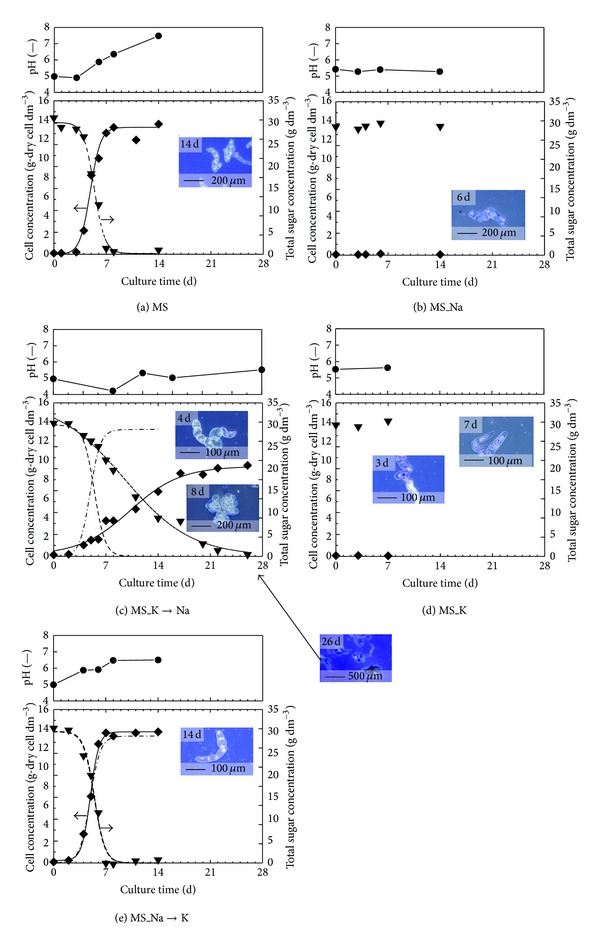
Time courses of the pH, cell, and total sugar concentrations during cultivation using a basal MS medium (a) and modified MS media represented by MS_Na (b), MS_K→Na (c), MS_K (d), and MS_Na→K (e). The dot-dash and dash lines in (c) and (e) show the growth and total sugar profile from (a), results of the basal MS medium cultivation.

Figure 2 shows the time courses of the pH, cell, and total sugar concentrations during cultivation using the basal MS medium and modified MS media. In Figures 2(a), 2(c), and 2(e), solid and broken lines indicate the fitting results for the cell growth and total sugar concentration using (1), respectively. To compare the growth curve of the MS_K→Na or MS_Na→K cultivation with the basal MS cultivation, the results of the growth curve of the basal MS medium cultivation were drawn in Figures 2(c) and 2(e) by using dot-dash line. The sugar consumption curve was also drawn by using dash line in Figures 2(c) and 2(e).

In the basal MS cultivation, the inoculum suspension cell was grown after a few days and the growth was stopped by 9 days of cultivation (Figure 2(a)). The suspension cells in the MS_Na and MS_K media were not grown. Therefore, the cultivation using the diluted media to a half ionic intensity of these media was tried; however, the cells were not grown in the diluted media (data not shown). Almost all the metallic salts were replaced with potassium salt or sodium salt in these media; the cells could not grow in the unbalanced salt conditions. In particular, dead cells were observed after 7 days of cultivation in the MS_K medium (Figure 2(d)). In the medium which replaced potassium salt with sodium salt, MS_K→Na, cell growth was observed. However, the growth and sugar consumption rates were lower than the case of the basal MS cultivation (Figure 2(c)). On the other hand, in the case of the MS_Na→K medium which replaced sodium salt with potassium salt, inhibition of the cell growth was not observed. In the potassium ion, potassium chloride is often substituted for sodium chloride in food; this might play an important role in activating the living cells. The activity of the living cells in food seems to be significantly influenced by a kind of used salt. When sodium-reduced foods are made by using potassium salt, attention should be paid about these influences on activity of the living cells.

### 3.3. Detection of Activated Cells for the High-Throughput Spectroscopic Method

**Figure 3:**
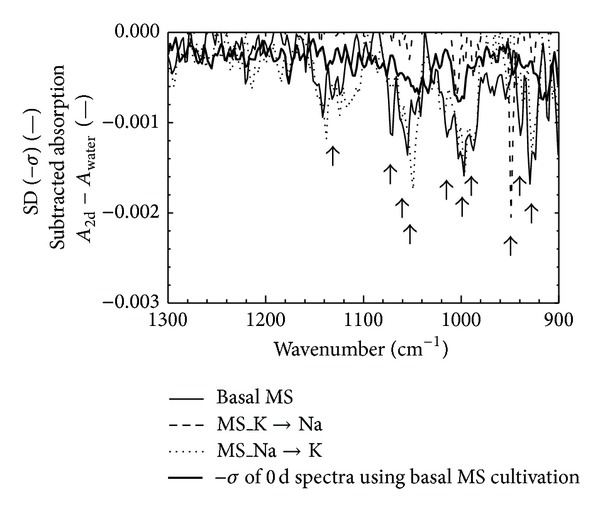
Subtracted spectra at 2 days of cultivation which were obtained after spectral subtraction of 0 days of absorption and the standard deviation (SD) of the six spectra of the 0 days of cultivation using a basal MS medium.

Several models for different states of the contaminated cells were constructed by changing the salt condition as shown in Figure 2. It is considered that the spectral feature change as shown in Figure 1 reflects the cell growth and the sugar consumption kinetic phenomena. Therefore, checking the spectral pattern might be a useful method for monitoring the activity of the contaminated suspension cells.  Figure 3 shows the subtracted spectra at 2 days of cultivation, which were obtained after spectral subtraction of the 0 days of absorption. The standard deviation (SD) of the six spectra of the 0 days of cultivation using a basal MS medium was also displayed in Figure 3. When the SD value was set at the threshold for recognition of the spectral changes, the start of the cell activation could be detected at 2 days in the basal MS, the MS_K→Na, and the MS_Na→K cultivations. The cell growth was not started at 2 days as shown in Figure 2; however, the cell activation (start of the sugar metabolism) could be detected by checking the spectral feature changes. On the subtracted spectra, the absorptions that were larger than the SD value were caused by the structure of the sugar. The contaminated cells proliferated in the food model, however, the cells did not grow for a while just after the contamination. During the induction period, the cells prepare for the growth although the cell growth was not observed; the evidence shows that the cell activities may be caught as the spectral feature changes. In fact, sucrose in the culture media is hydrolyzed to glucose and fructose by the enzymes in the cells before the growth, and the formed glucose and fructose are consumed for cell growth [26]. This hydrolyzation reaction means that the start of the sugar metabolism and the reaction is begun before the cell growth. These results suggested that the measurement and recode of the time courses of the spectral data were useful to check the microbial contamination and the evaluation of the cell activity in food. The rough grasp of the behavior of the spectral change represented in Figure 3 is important for the high-throughput spectroscopic method. Spectroscopic analysis is a powerful tool for the simultaneous measurement of the metabolite because theoretically all the information about the organic matters in food is contained in the IR spectrum. Therefore, more detailed analysis for each metabolic material of the cells might be possible by using spectral data.

### 3.4. Sugar Metabolic Kinetics

**Figure 4:**
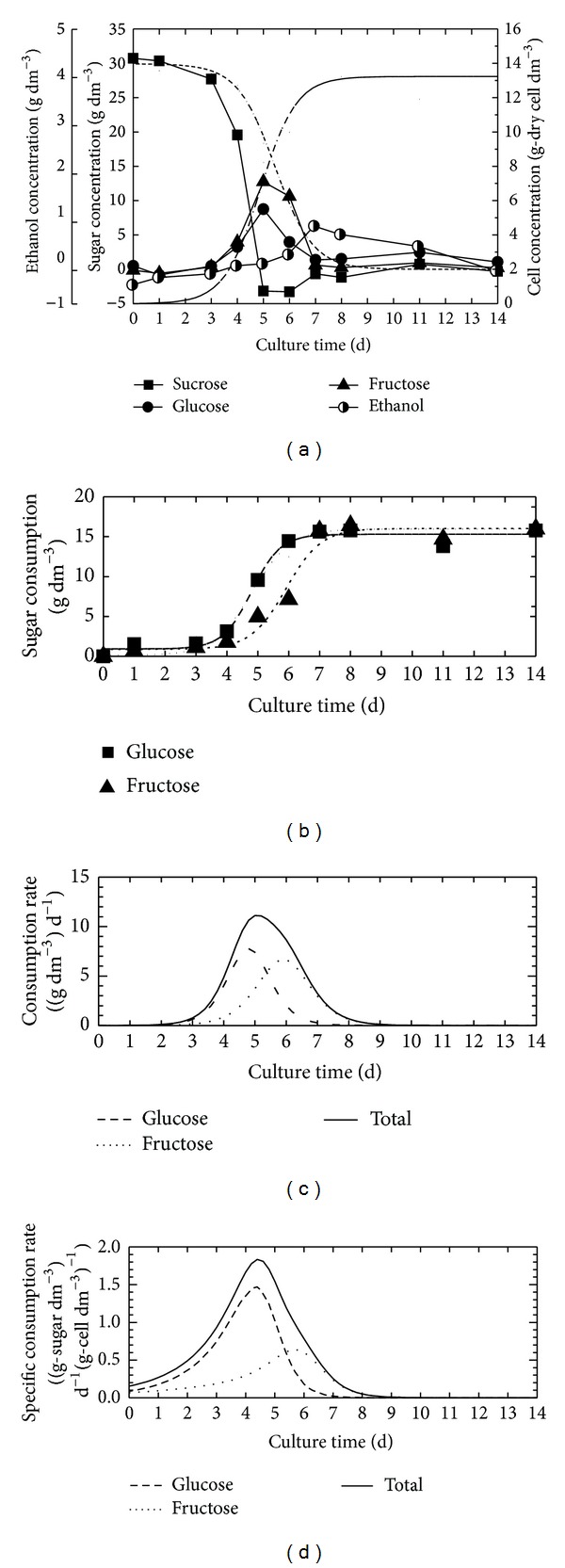
Time courses of sugar and ethanol concentrations (a), amount of the glucose and fructose consumptions (b), consumption rate (c), and specific consumption rate (d) of basal MS cultivation.

Figure 4(a) shows the time courses of the sucrose, glucose, fructose, and ethanol concentrations for the basal MS cultivation based on the absorbance of the FT-IR/ATR spectrum at 1,055, 1,036, 1,065, and 1045 cm^−1^. The details of several of the metabolite concentrations could be analyzed by the spectroscopic method simultaneously. For analysis the detailed metabolism process of the sugar, the time courses of the glucose and the fructose consumptions, was calculated by the material balance of the sucrose, glucose, and fructose by the assumption that the suspension cells consume sucrose after hydrolysis. The amount of the glucose and the fructose consumption could be expressed by the logistic function and the consumption rates of glucose and fructose were calculated by differentiating the logistic function with the cultivation time (Figures 4(b) and 4(c)). In addition, the specific consumption rate was calculated by dividing the consumption rate in the cell concentration (Figure 4(d)). The sugar metabolic kinetics could be investigated more clearly by these calculation results.

**Figure 5:**
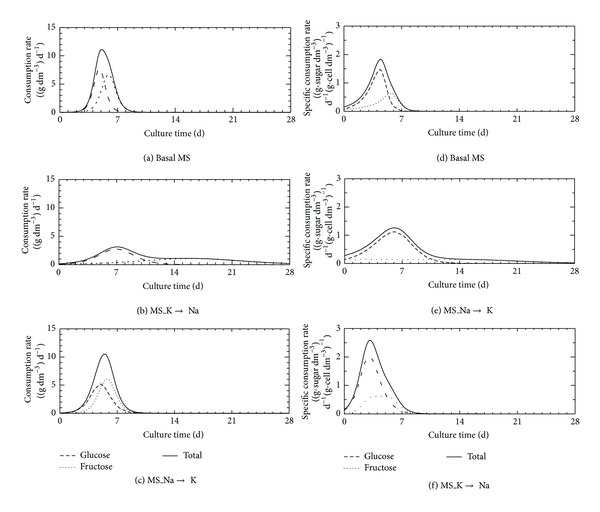
Time courses of consumption and specific consumption rates of glucose, fructose, and total sugar in cultivations using basal MS ((a), (d)), MS_K→Na ((b), (e)), and MS_Na→K ((c), (f)) media.

Figure 5 shows the consumption and specific consumption rates of the glucose, fructose, and the total sugar in the cultivations using basal MS, MS_K→Na, and MS_Na→K media. In all cases, the consumption of glucose was observed earlier than fructose. This result suggested that the sugar metabolism of cells was fundamentally not changed even if a kind of the salt changed in the media. However, the peak time and rate (height and the shape of the peak) were different under each salt condition. In the case of the MS_K→Na cultivation, two peaks were observed because the consumption of fructose was delayed. In addition, consumption and specific consumption rates of fructose of the MS_K→Na cultivation were considerably slower than these of the other cases. In the case of the MS_Na→K cultivation, glucose consumption and specific consumption rates were slightly slower than those of the cases of fructose. Although the cell growth curve of the MS_Na→K cultivation was almost the same as that of the basal MS cultivation (Figure 2(e)), the sugar metabolism seems to be slightly different. Sugar metabolism of the cells might be changed by replacement of the kind of the salt in the medium. Therefore, we calculated the consumption and specific consumption rates of the sugar based on the nondimensional culture time of the cells to chancel the differences of the cell growth profile in each cultivation.

**Figure 6:**
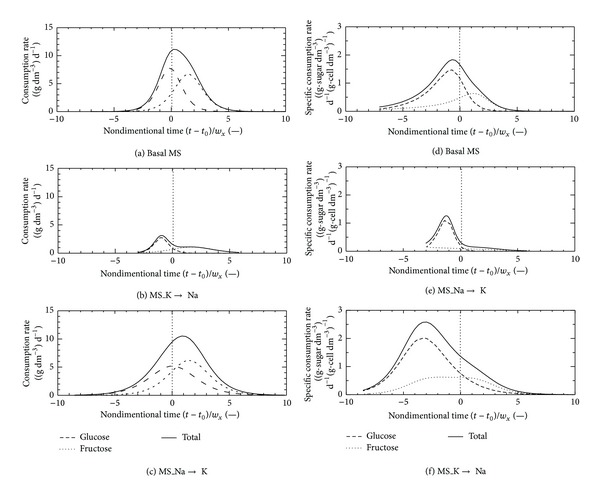
Time behavior of nondimensional time of consumption and specific consumption rates of glucose, fructose, and total sugar in cultivations using basal MS ((a), (d)), MS_K→Na ((b), (e)), and MS_Na→K ((c), (f)) media.

Figure 6 shows the kinetics of the sugar consumption characteristics based on the cell growth stage. In Figure 6, salt influence on sugar consumption by suspension cells was observed more clearly. In the basal MS cultivation, the peak of the consumption and specific consumption rates were observed at around nondimensional time zero. Both the growth and the sugar consumption progressed at approximately the same time (Figures 6(a) and 6(d)). On the other hand, small and broad peaks were observed in the negative period of the nondimensional time in the case of the MS_K→Na cultivation (Figures 6(b) and 6(e)). In our previous study, various saccharides such as glucose, fructose, galactose, mannose, sucrose, trehalose, and maltose were used as carbon sources for the TBY-2 cell cultivation. The peak times on the nondimensional time were almost the same even if the added sugar was changed in the basal MS salt medium except mannose and galactose which are known to be toxic to plant growth [23]. The influence of salt on the sugar consumption by the suspension cells was more significant than that in the case of the sugar change. In addition, sugar was consumed for preservation by the living cells although the cell growth was suppressed by changing the salt conditions. It seems that the sugar consumption was carried out before the cell growth, but the consumption rate was lower than the basal MS cultivation in the case of the MS_K→Na cultivation.

In the MS_Na→K cultivation, the cell growth and the sugar consumption profiles were almost the same as those of the basal MS cultivation; however, drastic changes in the sugar kinetics were observed. In particular, the peak of the sugar specific consumption rate was shifted to the negative period of the nondimensional time more clearly. However, the cell growth was not suppressed in this situation. The reason for this phenomenon is not clear, but the behaviors of the consumption of the glucose and the fructose were slightly changed in comparison with the case of the basal MS cultivation. The sugar specific consumption rate of the MS_Na→K cultivation was faster than that of the basal MS cultivation. In general, most living cells are easy to utilize glucose than fructose. In all cases, the clear peak of the glucose consumption and specific consumption rates were observed. Therefore, the sugar metabolism of glucose will not be influenced under the changing salt conditions in medium. The peak shape of the fructose consumption rate depended on the kind of salt in the medium, accordingly it may have given the change for sugar metabolism behavior. In particular, cell growth in the early period of the cultivation was significantly suppressed in the MS_K→Na cultivation.

The living cells activity in fresh food may act to prevent microbial contamination and to suppress the growth of the contaminated microorganism. Sodium-restricted food (potassium chloride is used as a food additive for the replacement of sodium chloride) will be advantageous to keeping the living cells activity in the food. The kind of salt added in the food influenced the cell activation and the potassium ions play an important role in the living plant cells.

Physiological influences of the cations for plants were reported in several studies [29–31] though the reports for suspension cells are extremely few. In these reports, several molecule components (ion channels) have been shown to contribute to potassium uptake into plant roots [29]. As a result, potassium ion counteracts influence of sodium ions for the plant because the sodium ion is toxic to most plants at high millimolar concentrations [30]. Furthermore, plants maintain a high concentration of potassium ion and a low concentration of sodium ion in cell cytosol under condition of salt stress [31]. Although the situation is different from the differentiated cells which construct the plants, the salt influence on sugar consumption by the living cells might be appropriately evaluated by the spectroscopic analysis presented in our study. In addition, all the information about the organic matters in food is theoretically contained in spectrum. In the future, the detailed spectroscopic information might be applied to determine the characteristics of the contaminated cells in the food even if the evidence is obtained indirectly [32].

## 4. Conclusion

The activity of the cell which invaded the food model was changed by the added salt changing, using the modified MS medium. In addition, the cell activation (start of the sugar metabolism) could be detected by checking if the spectral feature changed before the start of the cell growth. The measurement and recode of the subtracted spectral data were useful to checking the microbial contamination and the evaluation of the cell activity in food for risk management of the contamination. The rough grasp of the behavior of the spectral change is important for the high-throughput spectroscopic method. The spectral information containing the metabolite components of the activated cells and detailed analysis of the sugar consumption kinetics of the suspension cells could be investigated by spectroscopic method. Both the simple and detailed analyses based on the spectroscopic method presented in this study might be useful for evaluation of the activity of living cells in the food. The rough grasp of the behavior of the spectral change is important for the high-throughput spectroscopic method. Checking the spectral feature changes is useful for the daily check of the activation of the contaminated cells to avoid the contamination. The spectroscopic analysis will be greatly useful if the detailed information of the contaminated cells is necessary. Thus, the proposed methods will be applied to the risk management of various situations of microbial contamination in food.

## Figures and Tables

**Table 1:** Composition of basal MS medium and modified MS media.

Components	Basal MS	MS (K→Na)	MS (Na→K)	MS (Na)	MS (K)
Ammonium nitrate	1.65 × 10^3^	1.65 × 10^3^	1.65 × 10^3^	1.65 × 10^3^	1.65 × 10^3^
Boric acid	6.20	6.20	6.20	6.20	6.20
Potassium nitrate	1.90 × 10^3^		1.90 × 10^3^		1.90 × 10^3^
Sodium nitrate		1.56 × 10^3^		1.56 × 10^3^	
Potassium dihydrogen phosphate	1.70 × 10^2^		1.70 × 10^2^		1.70 × 10^2^
Sodium dihydrogen phosphate		1.50 × 10^2^		1.50 × 10^2^	
Potassium iodide	8.30 × 10^−1^		8.30 × 10^−1^		8.30 × 10^−1^
Sodium iodide		7.50 × 10^−1^		7.50 × 10^−1^	
Disodium ethylenediaminetetraacetate	3.73 × 10^1^	3.73 × 10^1^		3.73 × 10^1^	
Dipotassium ethylenediaminetetraacetate			4.05 × 10^1^		4.05 × 10^1^
Disodium molybdate dihydrate	2.50 × 10^−1^	2.50 × 10^−1^		2.50 × 10^−1^	
Dipotassium molybdate dihydrate			2.50 × 10^−1^		2.50 × 10^−1^
Calcium chloride dihydrate	4.40 × 10^2^				
Cobalt chloride hexahydrate	2.50 × 10^−2^				
Sodium chloride				1.75 × 10^2^	
Potassium chloride					2.23 × 10^2^
Magnesium sulfate heptahydrate	3.70 × 10^2^	3.70 × 10^2^	3.70 × 10^2^		
Manganese sulfate monohydrate	2.23 × 10^1^	2.23 × 10^1^	2.23 × 10^1^		
Zinc Sulfate heptahydrate	8.60	8.60	8.60		
Copper(II) sulfate pentahydrate	2.50 × 10^−2^	2.50 × 10^−2^	2.50 × 10^−2^		
Iron(II) sulfate heptahydrate	2.78 × 10^1^	2.78 × 10^1^	2.78 × 10^1^		
Sodium sulfate				2.47 × 10^2^	
Potassium sulfate					3.01 × 10^2^
Glycine	2.00	2.00	2.00	2.00	2.00
Myoinositol	1.00 × 10^2^	1.00 × 10^2^	1.00 × 10^2^	1.00 × 10^2^	1.00 × 10^2^
Thiamin hydrochloride	1.00 × 10^−1^	1.00 × 10^−1^	1.00 × 10^−1^	1.00 × 10^−1^	1.00 × 10^−1^
Pyridoxine hydrochloride	5.00 × 10^−1^	5.00 × 10^−1^	5.00 × 10^−1^	5.00 × 10^−1^	5.00 × 10^−1^
Nicotinic acid	5.00 × 10^−1^	5.00 × 10^−1^	5.00 × 10^−1^	5.00 × 10^−1^	5.00 × 10^−1^
2,4-Dichrorophenoxy acetic acid	2.00 × 10^−1^	2.00 × 10^−1^	2.00 × 10^−1^	2.00 × 10^−1^	2.00 × 10^−1^
Sucrose	3.00 × 10^4^	3.00 × 10^4^	3.00 × 10^4^	3.00 × 10^4^	3.00 × 10^4^

Unit: mg dm^−3^.
